# Concurrent Chikungunya and Dengue Virus Infections during Simultaneous Outbreaks, Gabon, 2007

**DOI:** 10.3201/eid1504.080664

**Published:** 2009-04

**Authors:** Eric M. Leroy, Dieudoné Nkoghe, Benjamin Ollomo, Chimène Nze-Nkogue, Pierre Becquart, Gilda Grard, Xavier Pourrut, Rémi Charrel, Grégory Moureau, Angélique Ndjoyi-Mbiguino, Xavier De Lamballerie

**Affiliations:** Centre International de Recherches Médicales de Franceville, Franceville, Gabon (E.M. Leroy, D. Nkoghe, B. Ollomo, C. Nze-Nkogue, P. Becquart, G. Grard, X. Pourrut); Université de la Méditerranée, Marseille, France (R. Charrel, G. Moureau, X. De Lamballerie); Université des Sciences de la Santé, Libreville, Gabon (A. Ndjoyi-Mbiguino)

**Keywords:** Vector-borne infections, viruses, chikungunya, dengue, Aedes albopictus, arbovirus, Africa, outbreaks, dispatch

## Abstract

An outbreak of febrile illness occurred in Gabon in 2007, with 20,000 suspected cases. Chikungunya or dengue-2 virus infections were identified in 321 patients; 8 patients had documented co-infections. *Aedes albopictus* was identified as the principal vector for the transmission of both viruses.

In the past 20 years, dengue virus (DENV) and chikungunya virus (CHIKV) have caused large and geographically wide ranging epidemics (*1*,*2*). Recent CHIKV outbreaks caused several million clinical cases in the Indian Ocean Islands and India (*3*). The virus has also dispersed to new regions, including Gabon in Africa and Italy (*4*,*5*). DENVs cause the most notable mosquito-borne viral disease in the world; ≈100 million infections occur annually worldwide, and the incidence has increased >30-fold in the past 50 years (*1*). Despite this tremendous expansion of both diseases, relatively few cases of Chikungunya fever have been reported in Africa (*3*,*6*,*7*), and few dengue-2 virus (DENV-2) epidemics have been reported (*8*,*9*). Simultaneous CHIKV and DENV-2 outbreaks have rarely been observed.

CHIKV and DENV-2 are frequently transmitted to humans by peridomestic *Aedes* mosquitoes. *Ae. aegypti* has been considered to be the principal vector in the urban transmission cycle, with *Ae. albopictus* and other anthropophilic *Aedes* spp. serving as secondary vectors (*10*,*11*). However, the actual situation is much more complex. First, *Ae. albopictus* was repeatedly shown to be a highly competent vector of CHIKV during the recent outbreaks in the Indian Ocean and Italy (*5**,**12*). Second, the overall distribution of *Aedes* mosquitoes is rapidly changing. Specifically, *Ae. albopictus* (the Asian tiger mosquito) has dispersed globally into new territories previously occupied by *Ae. aegypti*. As a consequence, the characteristics of DENV and CHIKV circulation and their outbreak dynamics are likely to be modified.

## The Study

**Figure 1:**
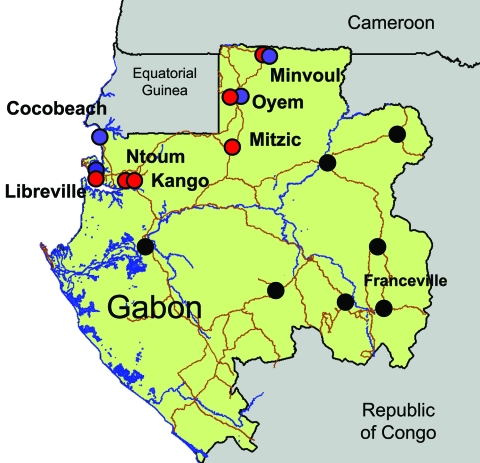
Chikungunya and dengue outbreaks in Gabon, 2007. Distribution of the outbreak and location of the 7 towns where suspected cases have been laboratory confirmed by using quantitative reverse transcription–PCR assay are shown. Chikungunya cases are represented by red circles, dengue cases by blue circles, and cases negative for the viruses by green circles. Testing methods are described in the footnote to the Table.

We report an arboviral outbreak that occurred in Gabon, Central Africa, from March through July 2007, which showed the unexpected extent of the spread of *Ae. albopictus* populations in peridomestic urban areas. We also describe its association with atypical epidemiologic characteristics such as the co-circulation of CHIKV and DENV-2 and the frequency of human co-infections. The outbreak centered on the capital of Gabon; peaked from April through May 2007, in the heat of the long wet season; and subsequently moved north, where the virus sequentially reached several small towns along the route to northern Gabon and Cameroon (Figure 1). The outbreak generated ≈20,000 cases. Patients with suspected cases exhibited a dengue-like syndrome, including fever, arthralgia, and asthenia. Conjunctival hemorrhage, maculopapular rash, headache, and vomiting were also observed in the most severe cases.

During the course of the outbreak, 773 early blood samples (i.e., obtained during the first week after the onset of the disease) were collected from febrile patients who visited identified medical health centers in Libreville and other towns in Gabon (Table). Samples were tested for the presence of various arboviral RNA genomes by using the TaqMan quantitative reverse transcription–PCR (qRT-PCR) technology and specific primers and probes (protocols available upon request to the corresponding author). Among these 773 patients, 275 and 54 were positive for CHIKV and DENV, respectively, during May and July 2007 (Table), with 8 cases of co-infections. Using a dengue serotype-specific qRT-PCR assay, we showed that all DENV patients were positive for serotype 2 (DENV-2). In all 7 towns investigated on the route from Libreville to Cameroon (530 km), both CHIKV and DENV-2 human cases were reported, except in Cocobeach where only laboratory DENV-2 confirmed cases were observed (Figure 1).

**Table:** Positive test results for CHIKV and DENV-2 among febrile patients, by town, Gabon, 2007*†

Towns	No. patients tested	No. CHIKV+	No. DENV-2+	No. CHIKV+/DENV-2+
Libreville	686	249	45	6
Ntoum	3	1	0	0
Kango	7	3	0	0
Mitzic	6	4	0	0
Oyem	45	15	2	1
Minvoul	7	3	1	1
Cocobeach	19	0	6	0
Total	773	275	54	8

*CHIKV, chikungunya virus; DENV-2, dengue-2 virus; +, positive. †RNA was extracted from 50 µL of plasma by using the ABI Prism 6100 Nucleic Acid PrepStation according to the manufacturer’s recommended procedures (Applied Biosystems, Foster City, CA, USA). Fifty-microliter aliquots of extracted RNA were then used in 100-µL High Capacity cDNA synthesis reactions according to the manufacturer’s instructions (Applied Biosystems). Finally, 10 µL of each cDNA reaction was then used as template for 50-µL quantitative PCRs that contained 200 nmol/L of probe and 900 nmol/L of each primer. The quantitative PCRs were then thermo-cycled in a 7500 Real-Time PCR system (Applied Biosystems) according to manufacturer’s recommended procedures. The probe used for the CHIKV, DENV, and DENV-2 assays were FAM-labeled with TAMRA quencher (Applied Biosystems).

To investigate this atypical scenario further, we analyzed 4,807 mosquitoes belonging to various species of *Aedes* (2,504 *Ae. albopictus*, 1,035 *Ae. aegypti*, 57 *Aedes sympsoni*), *Culex* (843 *Cx. quinquefasciatus*, 47 *Cx.* sp*.*), *Anopheles* (78 *An. gambiae*) and *Mansonia* (120 *M. africana*, 123 *M. uniformis*) in 15 different locations in Libreville where CHIKV or DENV-2 laboratory confirmed human cases were detected. Pools of 20 mosquitoes (constituted according to species and place of collection) were homogenized by using GenoGrinder 2000 (OPS Diagnostics, Bridgewater, NJ, USA) technology, and then tested for CHIKV and DENV-2 by qRT-PCR. We found that 7 and 3 groups of *Ae. albopictus* were positive for CHIKV and DENV-2, respectively, while no group containing other mosquito species was positive, indicating that *Ae. albopictus* was the only or at least the prominent vector of the 2 viruses.

**Figure 2:**
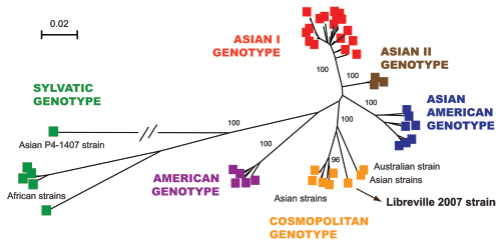
Phylogenetic relationships among dengue-2 virus (DENV-2) isolates based on full-length sequences (10,695 nt). A total of 85 DENV-2 genomes were compared with the human isolate obtained during the Gabon outbreak. Phylogeny was inferred by using neighbor-joining analysis. A neighbor-joining tree was constructed by using MEGA version 3.2 (www.megasoftware.net) with the Kimura 2-parameter corrections of multiple substitutions. Reliability of nodes was assessed by bootstrap resampling with 1,000 replicates. Branches are scaled according the number of substitutions per site, and the branch leading to the Thailand 94 strain was shortened for convenience. Bootstrap values are shown for major key nodes.

These data provide evidence for the presence of CHIKV and DENV in Gabon and for their transmission to humans by *Ae. albopictus*. These epidemiologic results also confirm our previous observation that CHIKV strains isolated during the Gabon outbreak in 2007 belong to the Central African lineage and harbor the A226V mutation as a result of adaptation to *Ae. albopictus* through a mechanism of evolutionary convergence (*4*). More surprisingly, our results show that the spread of this mosquito in an area previously occupied predominantly by *Ae. aegypti* (*13*) was accompanied by the simultaneous emergence and transmission of DENV-2. One DENV-2 strain (designated as Libreville 2007), isolated from 1 febrile patient by using E6 Vero cells was further characterized by full-length genome sequencing (10,695 nt). Phylogenetic analysis showed that the DENV-2 Gabon 2007 strain belongs to the cosmopolitan, rather than the sylvatic, genotype (Figure 2). This cosmopolitan genotype includes mainly Asian but also related strains isolated in India, Australia, Mexico, the Indian Ocean, and Africa (Uganda, Somalia, and Burkina Faso), presumably the result of travel to these remote locations by viremic patients or the transportation of commercial goods by ship.

## Conclusions

Taken together, these findings document CHIKV and DENV-2 co-circulation that resulted in large simultaneous outbreaks in regions where *Ae. albopictus* was shown to be the principal vector. Notably, we identified 8 patients with blood samples that tested positive for the presence of both CHIKV and DENV-2 genomes, indicating co-infection of these patients by both viruses. However, while unlikely, genetic exchanges between the 2 viruses, either by recombination or complementation, are not definitively excluded. Clinical examination of these patients (all adults, 5 women and 3 men) did not identify specific or severe symptoms, although given the limited number of cases and clinical and biologic investigations, this observation should be interpreted with caution.

Although the DENV cases were few, 8 of 48 (≈17%) DENV-2 positive patients from towns affected by the 2 outbreaks tested positive for CHIKV (Table). Extrapolation of this result suggests that the total number of DENV-2 patients who are superinfected with CHIKV is likely to be high, which suggests that DENV-2 infection is not the antagonist for a secondary CHIKV infection. In contrast, only ≈3% of CHIKV+ patients were also DENV-2+; however, the starting period of time of infection or the sequence of infection by the 2 viruses cannot be assessed.

Although concurrent infections of dengue and chikungunya have been reported (*14*), such DENV-2 and CHIKV co-infections have never been previously associated with transmission by *Ae. albopictus*. Our study therefore provides a disconcerting example of the unexpected epidemiologic patterns that may be associated with the dispersal of both vectors (*Ae. albopictus* and *Ae. aegypti*) and pathogenic arboviruses (such as DENV and CHIKV). *Ae. albopictus* mosquitoes are now present in several temperate countries of the Northern Hemisphere where, given the opportunity, they could cause future arboviral epidemics. The recent sustained indigenous transmission of CHIKV by *Ae. albopictus* in northern Italy (*5*) provides a potential warning of what might occur much more frequently in the future in Europe and even in North America. Introduction of DENV or CHIKV in these regions are likely to generate indigenous transmission by *Ae. albopictus*.
